# Retraction: LncRNA SNHG5 regulates the cell viability and apoptosis of glioma cells by the miR-1297/KPNA2 axis

**DOI:** 10.1039/d1ra90059e

**Published:** 2021-02-03

**Authors:** Laura Fisher

**Affiliations:** Royal Society of Chemistry Thomas Graham House, Science Park, Milton Road Cambridge CB4 0WF UK advances-rsc@rsc.org

## Abstract

Retraction of ‘LncRNA SNHG5 regulates the cell viability and apoptosis of glioma cells by the miR-1297/KPNA2 axis’ by Xueyuan Li *et al.*, *RSC Adv.*, 2020, **10**, 1498–1506, DOI: 10.1039/C9RA08693E

The Royal Society of Chemistry hereby wholly retracts this *RSC Advances* article due to concerns with the reliability of the data. The images in the article, and the raw data provided by the authors, were screened by an image integrity expert. The backgrounds to the individual panels of the raw data were sometimes almost blank, and had very large pixels, whereas the bands themselves had good resolution. It is therefore possible that the rows of bands were placed onto false backgrounds. Therefore, the raw data provided by the authors cannot be used as verifiable raw data to validate the published data.

Given the significance of the concerns about the validity of both the data in the article and the raw data provided by the authors, the findings presented in this paper are not reliable.

The authors have been informed but have not responded to any correspondence regarding the retraction.

Signed: Laura Fisher, Executive Editor, *RSC Advances*

Date: 19^th^ January 2021

## Supplementary Material

